# Effects of metformin on congenital muscular dystrophy type 1A disease progression in mice: a gender impact study

**DOI:** 10.1038/s41598-018-34362-2

**Published:** 2018-11-02

**Authors:** Cibely C. Fontes-Oliveira, Bernardo M. Soares Oliveira, Zandra Körner, Vahid M. Harandi, Madeleine Durbeej

**Affiliations:** Unit of Muscle Biology, Department of Experimental Medical Science, Lund University, Lund, Sweden

## Abstract

Congenital muscular dystrophy with laminin α2 chain-deficiency (LAMA2-CMD) is a severe muscle disorder with complex underlying pathogenesis. We have previously employed profiling techniques to elucidate molecular patterns and demonstrated significant metabolic impairment in skeletal muscle from LAMA2-CMD patients and mouse models. Thus, we hypothesize that skeletal muscle metabolism may be a promising pharmacological target to improve muscle function in LAMA2-CMD. Here, we have investigated whether the multifunctional medication metformin could be used to reduce disease in the *dy*^2*J*^*/dy*^*2J*^ mouse model of LAMA2-CMD. First, we show gender disparity for several pathological hallmarks of LAMA2-CMD. Second, we demonstrate that metformin treatment significantly increases weight gain and energy efficiency, enhances muscle function and improves skeletal muscle histology in female *dy*^*2J*^*/dy*^*2J*^ mice (and to a lesser extent in *dy*^*2J*^*/dy*^*2J*^ males). Thus, our current data suggest that metformin may be a potential future supportive treatment that improves many of the pathological characteristics of LAMA2-CMD.

## Introduction

Mutations in the *LAMA2* gene encoding laminin α2 chain cause congenital muscular dystrophy with laminin α2 chain-deficiency (LAMA2-CMD), a very severe muscle disorder. Under normal conditions, laminin α2 chain forms the heterotrimeric protein laminin-211 (together with laminin β1 and γ1 chains) and this extracellular matrix protein is highly expressed in the basement membranes of muscle and Schwann cells. Laminin α2 chain is either completely or partially absent in LAMA2-CMD and the clinical manifestations include profound hypotonia at birth, widespread muscle weakness, proximal joint contractures, inability to stand and walk, scoliosis, dysmyelinating neuropathy and white matter abnormalities^1^. At the histological level the skeletal muscle pathology comprises muscle fiber size variation, the presence of regenerating and necrotic fibers, vast inflammation and extensive proliferation of connective tissue^2^.

In order to obtain novel insights into the molecular mechanisms underlying LAMA2-CMD, we previously performed transcriptional and proteomic profiling of affected skeletal muscles from LAMA2-CMD mice. A majority of the differentially expressed genes and proteins were found to be involved in various metabolic processes^3,4^. Subsequently, we demonstrated functional bioenergetic impairment with reduced mitochondrial respiration and a compensatory upregulation of glycolysis in human LAMA2-CMD muscle cells^5^. Thus, from these studies, we concluded that skeletal muscle metabolism may be a promising pharmacological target to improve muscle function in LAMA2-CMD patients.

Metformin, a biguanide derived from *Galega officinalis*, has been used for more than 50 years to treat type II diabetes^6^. Despite long clinical use, its mechanisms of action still remain obscure, but several studies have demonstrated the effectiveness of metformin in skeletal muscle. For example, it was recently demonstrated that six weeks of metformin treatment increased expression of non-metabolic and metabolic-related genes in adipose and muscle tissue in old humans^7^. Furthermore, positive non-metabolic effects in exercised *mdx* mice (a mouse model of Duchenne muscular dystrophy) were observed after 20 weeks of metformin treatment, with improved skeletal muscle histopathology and force^8^. Moreover, metformin has been shown to protect skeletal muscle from cardiotoxin-induced degeneration^9^, increase physical performance in sedentary mice^10^ and enhance *Pgc1*α in dystrophin-deficient *mdx* muscle^11^. Also, an open-label proof-of-concept study demonstrated improved muscle function in four out of five ambulatory Duchenne muscular dystrophy patients treated with L-arginine and metformin^12^. Therefore, we reasoned that metformin might improve muscle function and delay disease progression in LAMA2-CMD. Hence, in this study, we have treated *dy*^*2J*^*/dy*^*2J*^ mice with metformin. *Dy*^*2J*^*/dy*^*2J*^ mice exhibit a mutation in the N-terminal domain of laminin α2 chain causing a laminin polymerization defect^13^. Consequently, *dy*^*2J*^*/dy*^*2J*^ mice present a relatively mild muscular dystrophy with the first symptoms appearing at around 3–4 weeks of age and *dy*^*2J*^*/dy*^*2J*^ mice typically live more than 6 months^2,13,14^. We demonstrate that *dy*^*2J*^*/dy*^*2J*^ mice treated with metformin display improved muscle structure and function. Importantly, we also analyzed the gender factor in the progression of the disease and demonstrate sex differences.

## Results

### Weight gain differences in male and female *dy*^*2J*^*/dy*^*2J*^ mice

**Figure 1:**
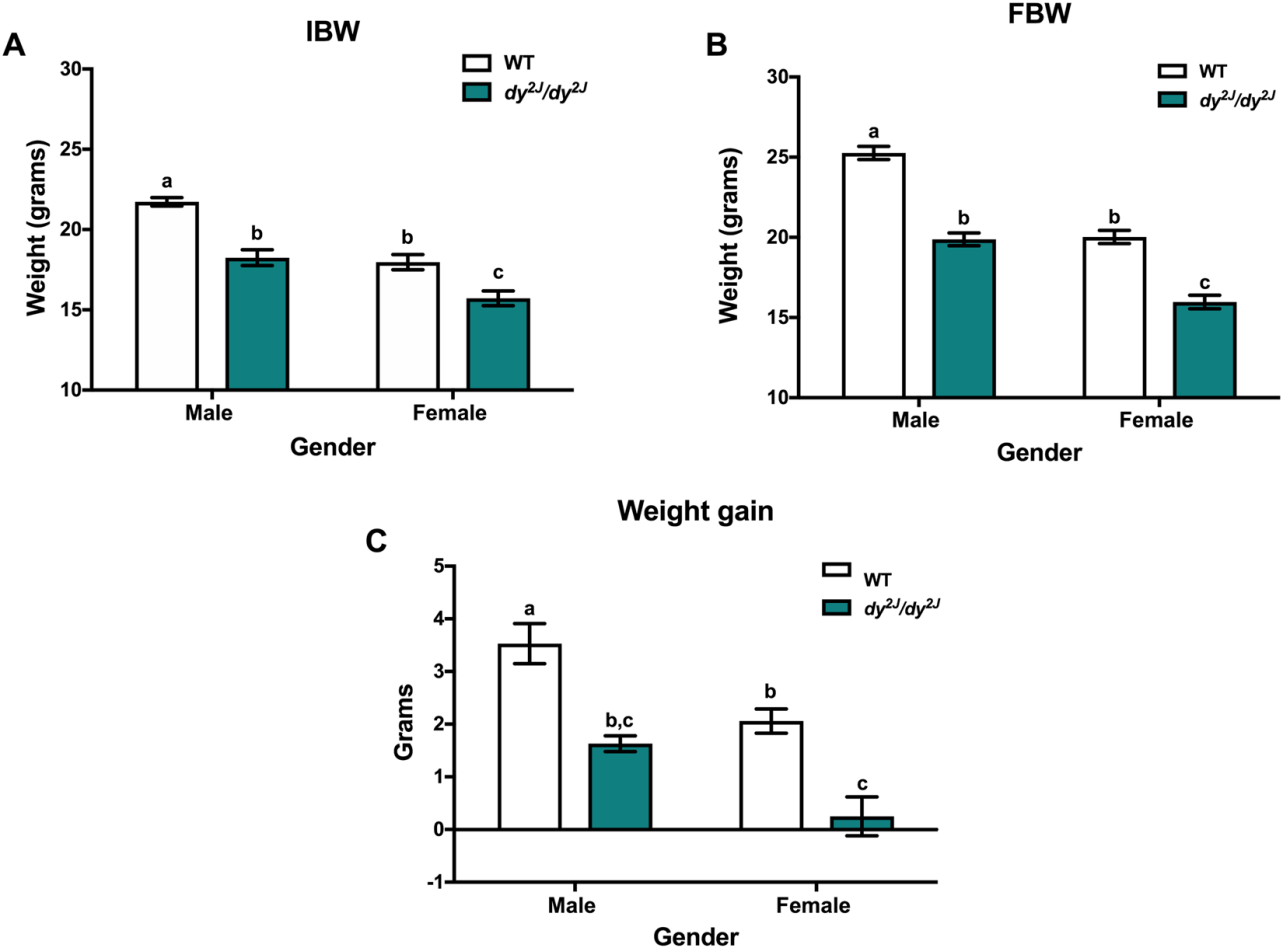
Weight differences between males and females during disease progression. (**A**) Initial body weight (IBW). (**B**) Final body weight (FBW). (**C**) Weight gain. Body weights were recorded when the animals were 6-weeks-old and monitored during four weeks. Results are expressed as mean ± SEM in 13 WT males, 7 WT females, 4 *dy*^*2J*^*/dy*^*2J*^ males and 5 *dy*^*2J*^*/dy*^*2J*^ females. IBW and FBW values are significantly different by two-way ANOVA for disease and gender both with p < 0.0001. Weight gain values are significantly different by two-way ANOVA for disease (p < 0.01) and gender (p < 0.001). Letters a, b and c were used to express the differences among groups and columns with the same letter are not significantly different.

To analyze the gender factor in the progression of the disease, males and females were separated in wild-type (WT) and *dy*^*2J*^*/dy*^*2J*^ groups. As expected, the initial body weight was significantly different between males and females in both WT and *dy*^*2J*^*/dy*^*2J*^ groups (Fig. 1A). The same trend was observed for the final body weight, with gender and disease significantly different when analyzed by two-way ANOVA (Fig. 1B). Notably, male *dy*^*2J*^*/dy*^*2J*^ mice displayed similar weights as female WT mice (Fig. 1A,B). When weight gain was compared between *dy*^*2J*^*/dy*^*2J*^ males and females, we noted that *dy*^*2J*^*/dy*^*2J*^ males gain around 85% more weight than *dy*^*2J*^*/dy*^*2J*^ females (Fig. 1C). Bearing this data in mind, metformin treatment was performed in males and females separately.

### Metformin treatment increases water intake and weight gain in female *dy*^*2J*^*/dy*^*2J*^ mice

We treated mice with metformin (250 mg/kg, daily oral gavage) during four weeks. The dose was chosen based on a small pilot study in which metformin at 100 mg/kg (daily oral gavage for four weeks) enhanced grip strength in female *dy*^*2J*^*/dy*^*2J*^ mice but the increase was not statistically different from untreated *dy*^*2J*^*/dy*^*2J*^ mice (data not shown).

**Figure 2:**
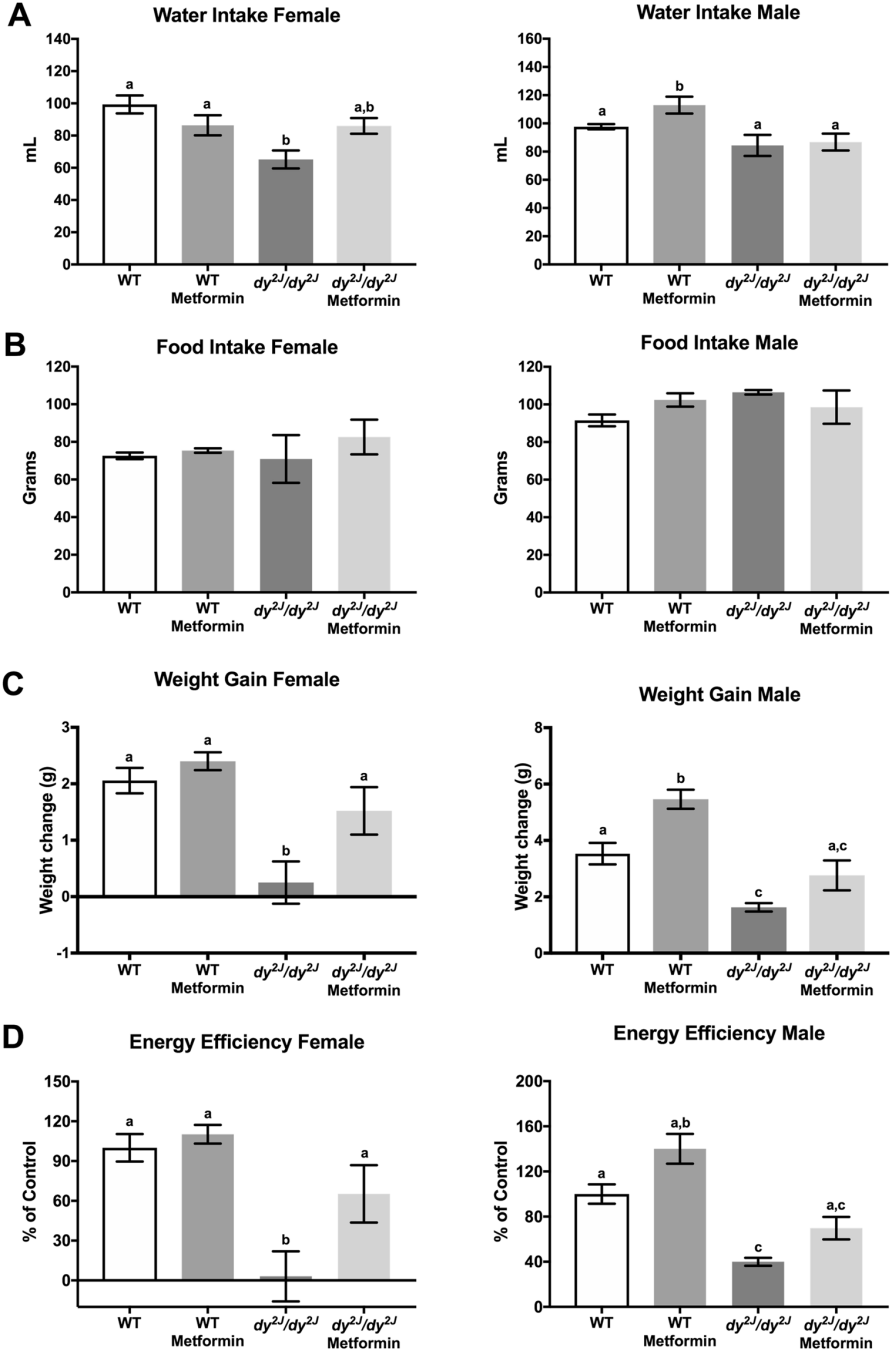
Differences between males and females in disease progression and response to metformin treatment. (**A**) Water intake expressed in milliliter (mL). (**B**) Food intake expressed in grams. The measurements refer to ingestion during the period of treatment. (**C**) Weight gain expressed in grams. (**D**) Energy efficiency expressed in percentage compared to WT control group, which is considered 100% efficient. WT control: females = 7, males = 13; WT metformin: females = 10, males = 6; *dy*^*2J*^*/dy*^*2J*^ control: females = 5, males = 4; *dy*^*2J*^*/dy*^*2J*^ metformin: females = 5, males = 5. Results are expressed as mean ± SEM. Statistical significance was assessed by one-way ANOVA followed by Bonferroni *post hoc* test. *p* < 0.05 values were considered as statistically significantly different. Letters a, b and c were used to express the differences among groups and columns with the same letter are not significantly different.

We first assessed the effects of metformin administration on water intake, food intake and weight gain. In *dy*^*2J*^*/dy*^*2J*^ females, we observed a 34% reduction of water intake compared with WT females (Fig. 2A). In contrast, water intake was not significantly different between *dy*^*2J*^*/dy*^*2J*^ and WT males (Fig. 2A). Interestingly, metformin treatment slightly increased water intake in *dy*^*2J*^*/dy*^*2J*^ females and also in WT males (Fig. 2A). Food intake was not significantly different between WT and *dy*^*2J*^*/dy*^*2J*^ mice, neither in males nor in females and metformin did not alter food intake in any of the groups (Fig. 2B).

An 87% reduction in weight gain was observed in *dy*^*2J*^*/dy*^*2J*^ females when compared with WT females whereas the reduction in males was around 54% (Fig. 2C). Metformin treatment had a positive effect in both *dy*^*2J*^*/dy*^*2J*^ females and males, but was more explicit in *dy*^*2J*^*/dy*^*2J*^ females, with levels statistically indistinguishable from those of WT mice (Fig. 2C). Accordingly, energy efficiency (calculated based on the weight gain and energy intake along the experimental period^15^) was positively affected by metformin with a six-fold increase (statistically significant) in *dy*^*2J*^*/dy*^*2J*^ females and a 1.7-fold increase (non-significant) in *dy*^*2J*^*/dy*^*2J*^ males (Fig. 2D).

### Metformin treatment does not enhance muscle weight but augments forelimb grip strength in female *dy*^*2J*^*/dy*^*2J*^ mice

**Figure 3:**
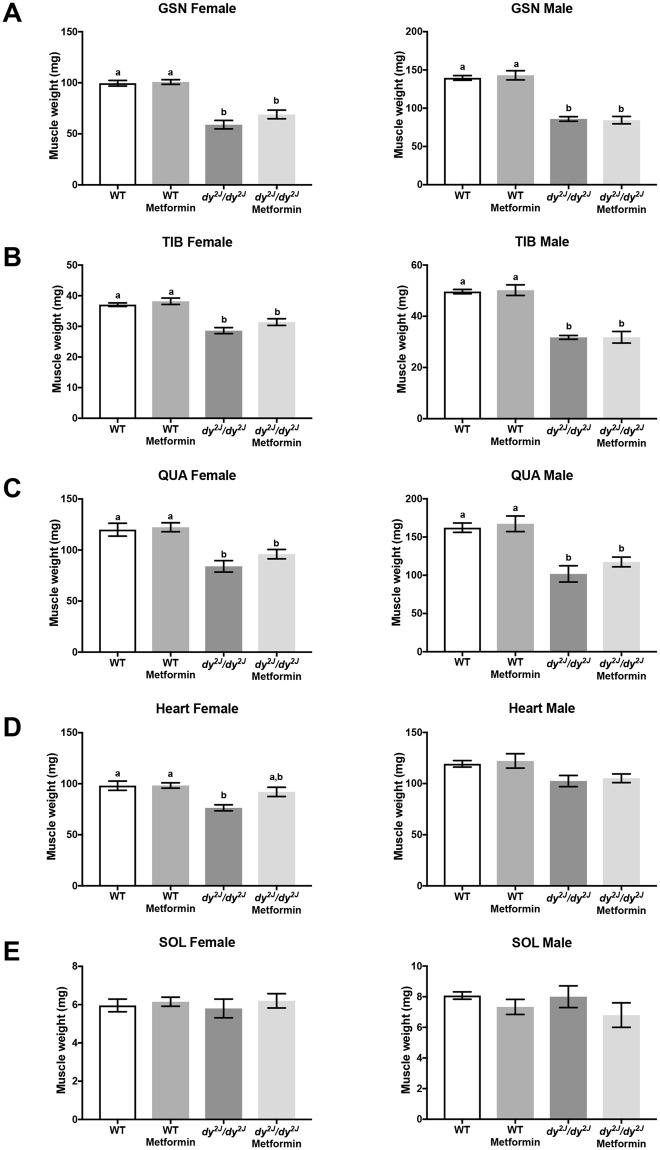
Muscle and heart weights. (**A**) Gastrocnemius muscle (GSN). (**B**) Tibialis anterior muscle (TIB). (**C**) Quadriceps muscle (QUA). (**D**) Heart muscle. (**E**) Soleus muscle (SOL). Values are expressed in grams (weights collected after four weeks of treatment). WT control: females = 7, males = 13; WT metformin: females = 10, males = 6; *dy*^*2J*^*/dy*^*2J*^ control: females = 5, males = 4; *dy*^*2J*^*/dy*^*2J*^ metformin: females = 5, males = 5. Results are expressed as mean ± SEM. Statistical significance was assessed by one-way ANOVA followed by Bonferroni *post hoc* test. *p* < 0.05 values were considered as statistically significantly different. Letters a and b were used to express the differences among groups and columns with the same letter are not significantly different.

Next, we evaluated if the weight gain was associated with larger skeletal muscles in metformin-treated *dy*^*2J*^*/dy*^*2J*^ mice. A significant decrease in the weight of gastrocnemius (41% in females and 38% in males), tibialis anterior (23% in females and 36% in males) and quadriceps (30% in females and 37% in males) muscles was seen in *dy*^*2J*^*/dy*^*2J*^ mice (Fig. 3A–C). Also, heart muscle weight was significantly declined in *dy*^*2J*^*/dy*^*2J*^ mice (32% in females and 14% in males) (Fig. 3D). In contrast, there was no difference in the weight of *dy*^*2J*^*/dy*^*2J*^ soleus muscle (neither in males nor females) compared to WT soleus muscle (Fig. 3E). Metformin treatment had no major effect in improving muscle mass in female and male *dy*^*2J*^*/dy*^*2J*^ mice (Fig. 3), except for a slightly increased heart muscle weight in *dy*^*2J*^*/dy*^*2J*^ females (Fig. 3D).

**Figure 4:**
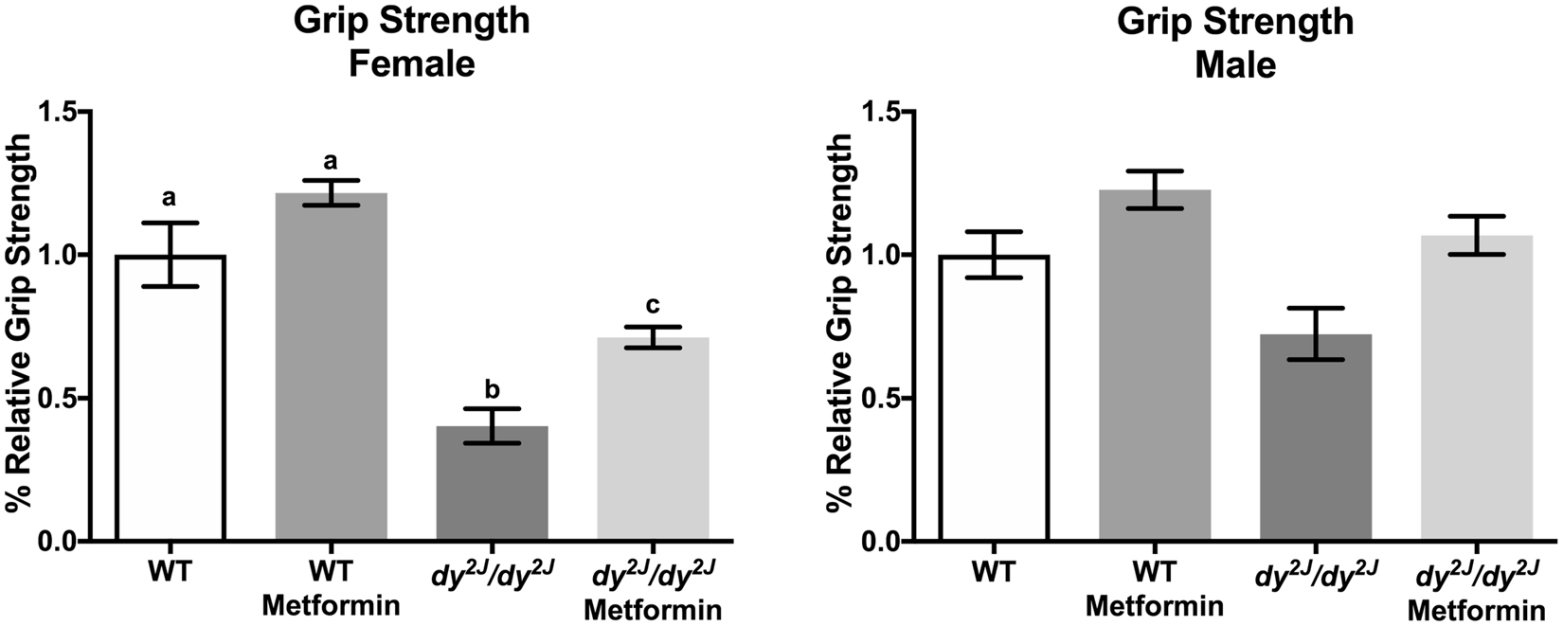
Relative forelimb grip strength. Calculations were done as force (KgF) divided by final body weight in grams. Results are expressed as % relative grip strength. WT control: females = 7, males = 13; WT metformin: females = 10, males = 6; *dy*^*2J*^*/dy*^*2J*^ control: females = 5, males = 4; *dy*^*2J*^*/dy*^*2J*^ metformin: females = 5, males = 5. Results are expressed as mean ± SEM. Statistical significance was assessed by one-way ANOVA followed by Bonferroni *post hoc* test. *p* < 0.05 values were considered as statistically significantly different. Letters a, b and c were used to express the differences among groups and columns with the same letter are not significantly different.

Yet, metformin significantly enhanced forelimb grip strength in *dy*^*2J*^*/dy*^*2J*^ mice. Forelimb grip strength was significantly reduced (about 70%) in *dy*^*2J*^*/dy*^*2J*^ females compared to WT females and metformin treatment approximately doubled the forelimb muscle strength (Fig. 4). Following this pattern, a 30% decrease in forelimb grip strength was observed in *dy*^*2J*^*/dy*^*2J*^ males when compared with WT (but this reduction was not statistically different). Furthermore, metformin treatment marginally improved forelimb grip strength in both WT and *dy*^*2J*^*/dy*^*2J*^ males (but again, the increase was not statistically different).

*Dy*^*2J*^*/dy*^*2J*^ mice display significant hindlimb paralysis and similar hindleg lameness was noted upon metformin treatment (data not shown). Also, exploratory locomotion was evaluated but metformin did not confer any beneficial effect in *dy*^*2J*^*/dy*^*2J*^ females or males (Supplemental Fig. S1).

### Skeletal muscle histology is improved in female *dy*^*2J*^*/dy*^*2J*^ mice

**Figure 5:**
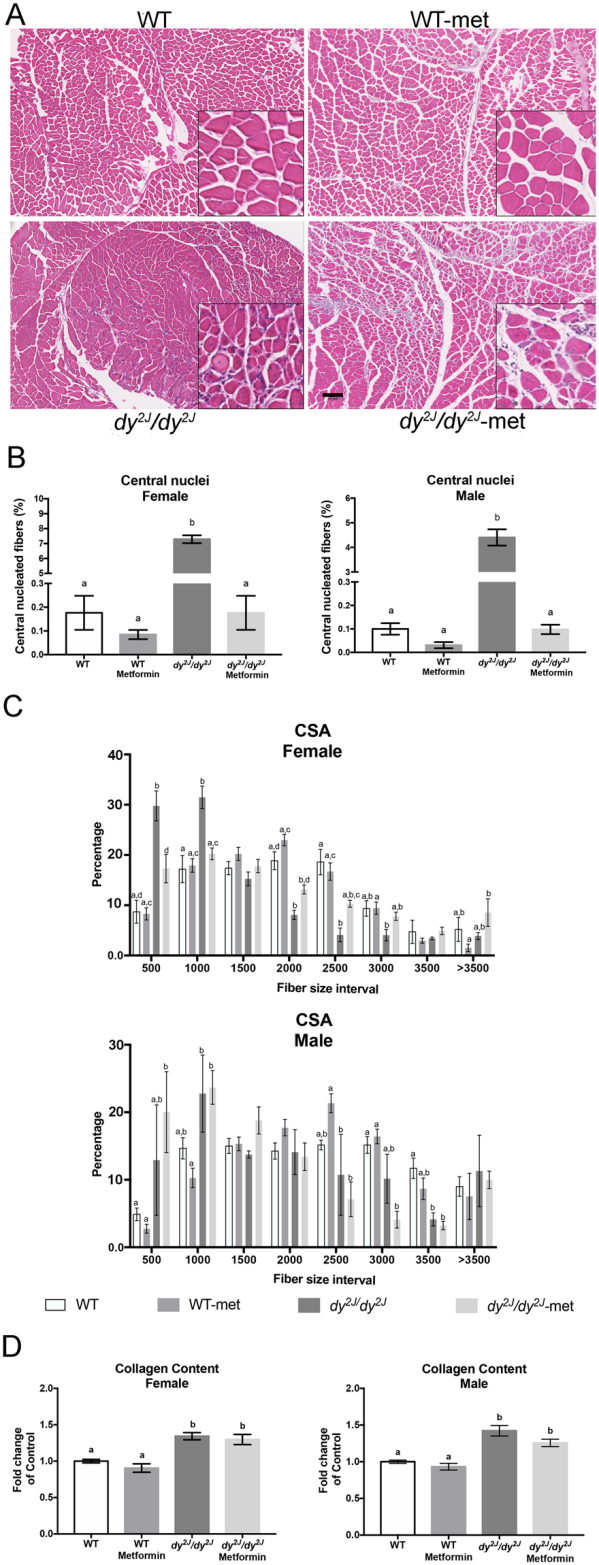
(**A**) Representative hematoxylin and eosin-stained quadriceps muscle sections from female mice. (**B**) Central nucleation in quadriceps muscle. (**C**) Cross-sectional area of quadriceps muscle cells. (**D**) Sirius red/fast green quantification in quadriceps muscle. WT control: females = 4, males = 4; WT metformin: females = 4, males = 4; *dy*^*2J*^*/dy*^*2J*^ control: females = 4, males = 4; *dy*^*2J*^*/dy*^*2J*^ metformin: females = 4, males = 3. Results are expressed as mean ± SEM. Statistical significance was assessed by one-way ANOVA followed by Bonferroni *post hoc* test. *p* < 0.05 values were considered as statistically significantly different. Letters a, b, c and d were used to express the differences among groups and columns with the same letter are not significantly different. Bar = 50 µm, Magnification = 4.9×.

A microscopic evaluation of H&E-stained quadriceps muscle sections revealed typical muscular dystrophy characteristics with fiber degeneration/regeneration (evidenced by central nucleation) and fiber size variability in *dy*^*2J*^*/dy*^*2J*^ mice (Fig. 5A). Central nucleation was significantly amplified in both *dy*^*2J*^*/dy*^*2J*^ females and males and metformin treatment reduced the number of fibers with centrally located nuclei to levels statistically indiscernible from those of WT mice (Fig. 5B). In *dy*^*2J*^*/dy*^*2J*^ female muscle, the percentage of fibers with cross sectional areas in the range of 500 to 1000 mm^2^ was robustly increased compared to WT counterparts. In contrast, the percentage of fibers between 2000 to 3000 mm^2^ was significantly decreased (Fig. 5C). Similarly, the percentage of fibers with cross sectional areas in the range of 500 to 1000 mm^2^ was increased compared to WT counterparts in *dy*^*2J*^*/dy*^*2J*^ male muscle and the percentage of fibers between 2500–3500 was decreased (although statistical significance was not reached in the 2500–3000 intervals) (Fig. 5C). Notably, metformin treatment caused a shift of fiber size distribution and normalized the fiber size and proportion in *dy*^*2J*^*/dy*^*2J*^ females (Fig. 5C) whereas metformin did not affect fiber size distribution in *dy*^*2J*^*/dy*^*2J*^ males (Fig. 5C).

Pathological fibrosis is also a typical feature of *dy*^*2J*^*/dy*^*2J*^ muscle and we quantified collagen content by sirius red and fast green staining. A similar collagen accumulation was noted in *dy*^*2J*^*/dy*^*2J*^ females and males, but metformin treatment did not reduce total collagen deposition (Fig. 5D).

**Figure 6:**
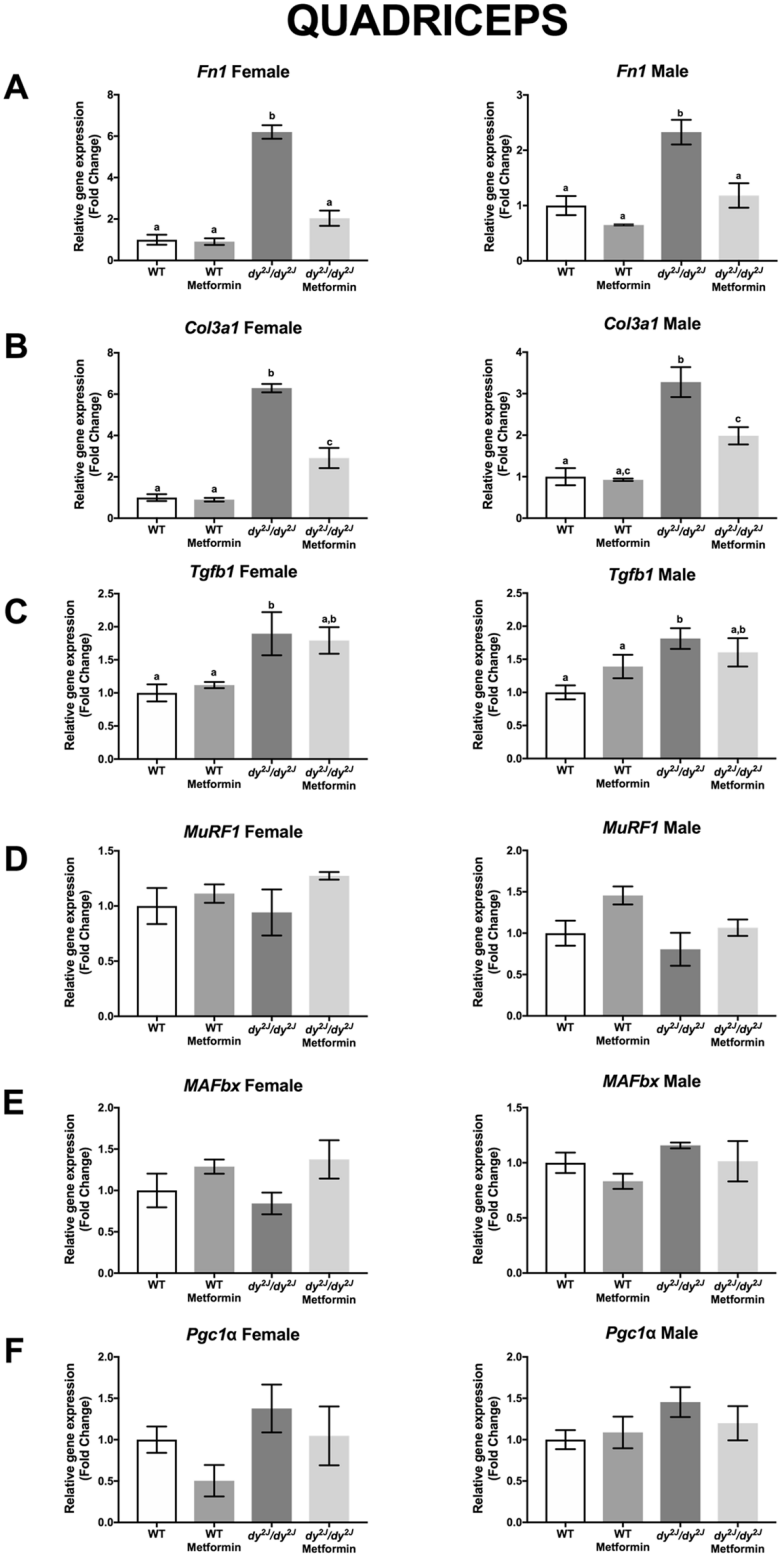
Gene expression measured by qPCR in quadriceps muscle. (**A**) Expression of *Fn1* encoding fibronectin. (**B**) Expression of *Col3a1* encoding the α1 chain of collagen III. (**C**) Expression of *Tgfb1* encoding TGF-β1. (**D**) Expression of *MuRF1* encoding muscle RING-finger protein-1. (**E**) Expression of *MAFbx* encoding muscle atrophy F-box protein. (**F**) Expression of *Pgc1a* encoding peroxisome proliferative activated receptor, gamma, coactivator 1α. n = 4–6 per group. Results are expressed as mean ± SEM and are expressed as fold change of WT. Statistical significance was assessed by one-way ANOVA followed by Bonferroni *post hoc* test. *p* < 0.05 values were considered as statistically significantly different. Letters a, b and c were used to express the differences among groups and columns with the same letter are not significantly different.

**Figure 7:**
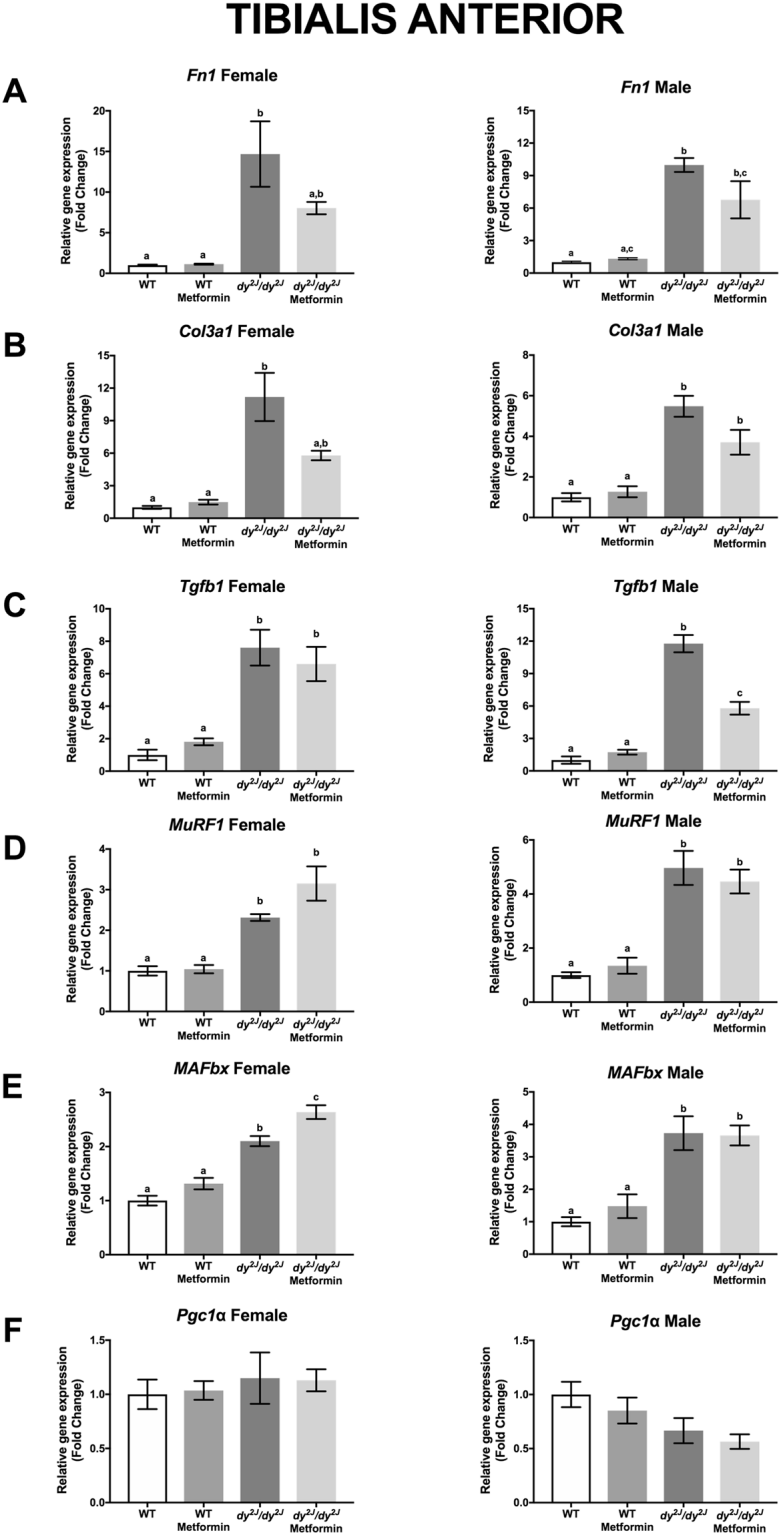
Gene expression measured by qPCR in tibialis anterior muscle. (**A**) Expression of *Fn1* encoding fibronectin. (**B**) Expression of *Col3a1* encoding the α1 chain of collagen III. (**C**) Expression of *Tgfb1* encoding TGF-β1. (**D**) Expression of *MuRF1* encoding muscle RING-finger protein-1. (**E**) Expression of *MAFbx* encoding muscle atrophy F-box protein. (**F**) Expression of *Pgc1a* encoding peroxisome proliferative activated receptor, gamma, coactivator 1α. n = 4–6 per group. Results are expressed as mean ± SEM and are expressed as fold change of WT. Statistical significance was assessed by one-way ANOVA followed by Bonferroni *post hoc* test. *p* < 0.05 values were considered as statistically significantly different. Letters a, b and c were used to express the differences among groups and columns with the same letter are not significantly different.

To further analyze connective tissue infiltration, we measured the relative gene expression of three different fibrosis-related genes; *Fn1* encoding fibronectin; *Col3a1* encoding the α1 subunit of collagen III and *Tgfb1* encoding TGF-β1 in quadriceps and tibialis anterior muscles. We found that the expression of *Fn1* was significantly increased in *dy*^*2J*^*/dy*^*2J*^ female and male quadriceps and tibialis anterior muscles. Notably, metformin normalized *Fn1* expression in female and male *dy*^*2J*^*/dy*^*2J*^ quadriceps muscle and reduced it in *dy*^*2J*^*/dy*^*2J*^ tibialis anterior muscle (Figs 6A and 7A). Similarly, *Col3a1* expression was significantly increased in *dy*^*2J*^*/dy*^*2J*^ female and male quadriceps and tibialis anterior muscles and metformin significantly reduced it in female and male *dy*^*2J*^*/dy*^*2J*^ quadriceps muscle (but to a lesser extent in *dy*^*2J*^*/dy*^*2J*^ tibialis anterior muscle) (Figs 6B and 7B). The expression of *Tgfb1*, on the other hand, remained enhanced in *dy*^*2J*^*/dy*^*2J*^ female and male quadriceps and tibialis anterior muscles upon metformin treatment (apart from a slight reduction in *dy*^*2J*^*/dy*^*2J*^ tibialis anterior) (Figs 6C and 7C). All in all, the gene expression analysis indicates that metformin affects some aspects of pathological fibrosis in *dy*^*2J*^*/dy*^*2J*^ skeletal muscle.

To further assess whether metformin impacts muscle atrophy, we measured the relative gene expression of *MurF1* encoding muscle RING-finger protein-1 and *MAFbx* encoding muscle atrophy F-box protein (two E3 ubiquitin ligases that are key regulators of muscle atrophy^16^. We found that expression of *MuRF1* and *MAFbx* is enhanced in 10-week-old *dy*^*2J*^*/dy*^*2J*^ tibialis anterior muscle from both males and females but not in corresponding quadriceps muscles and metformin did not alter the expression (except for increasing *MAFbx* expression in *dy*^*2J*^*/dy*^*2J*^ female tibialis anterior muscle) (Figs 6D,E and 7D,E).

Finally, we measured the expression of *Pgc1*α encoding peroxisome proliferator-activated receptor gamma coactivator 1α (a key regulator of mitochondrial metabolism) that we have shown to be decreased in LAMA2-CMD patient myotubes^5^. However, expression of *Pgc1*α was not altered in *dy*^*2J*^*/dy*^*2J*^ muscles and metformin did not change its expression either (Figs 6F and 7F).

**Figure 8:**
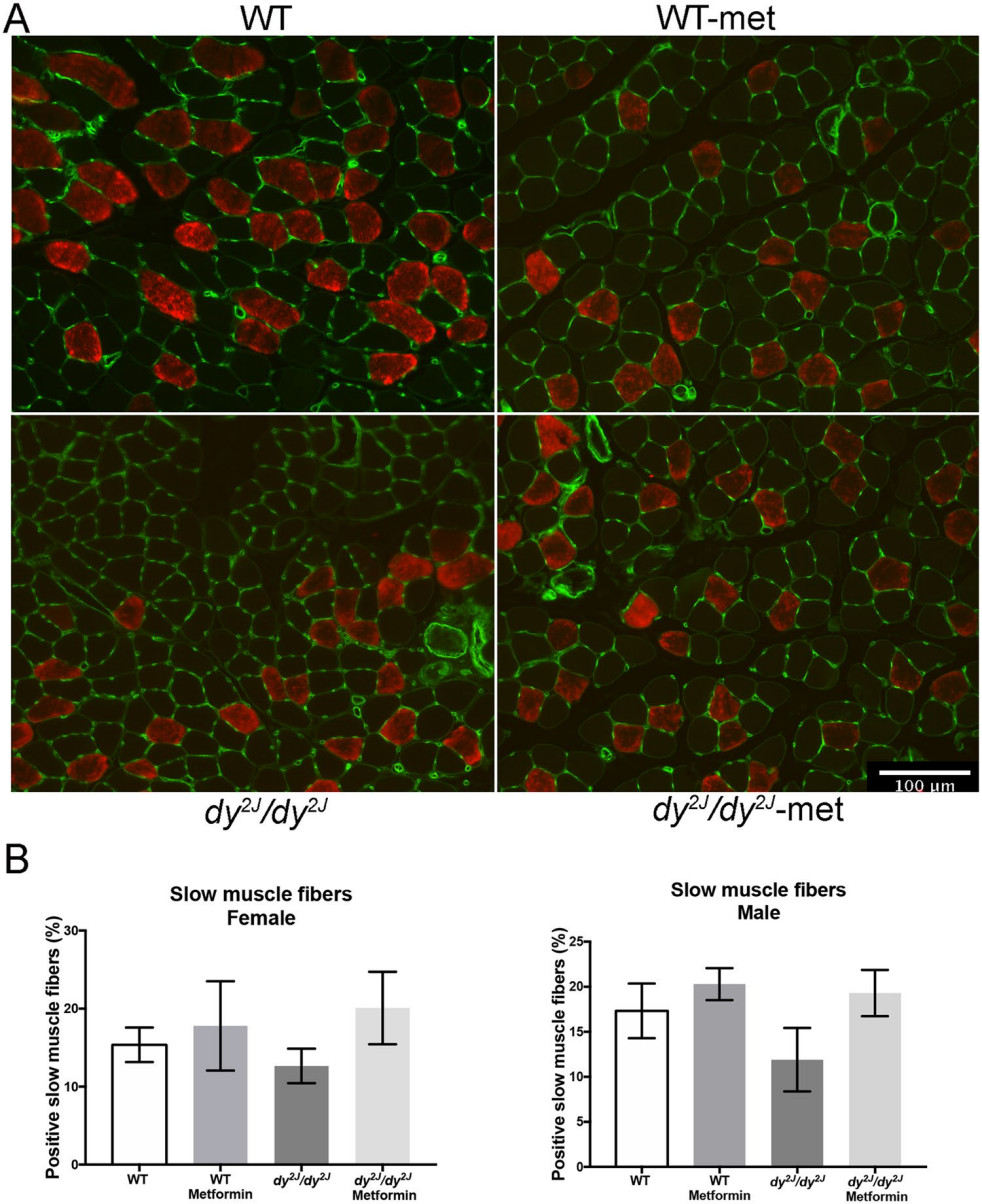
(**A**) Expression of MyHC-type 1 fibers (red) in female quadriceps muscle and membrane marker in green. (**B**) Values are expressed in percentage of positive slow muscle fibers (analyzed after four weeks of treatment). WT control: females = 3, males = 3; WT metformin: females = 3, males = 3; *dy*^*2J*^*/dy*^*2J*^ control: females = 3, males = 3; *dy*^*2J*^*/dy*^*2J*^ metformin: females = 4, males = 4. Results are expressed as mean ± SEM. Statistical significance was assessed by one-way ANOVA followed by Bonferroni *post hoc* test. Bar = 100 µm.

Lastly, to investigate whether metformin treatment affected fiber type composition, we analyzed the expression of myosin heavy chain (MyHC), type 1 that is expressed in slow twitch fibers (Fig. 8A). The number of fibers with positive staining was slightly reduced in *dy*^*2J*^*/dy*^*2J*^ mice (both females and males) compared to WT and metformin seemed to increase the percentage of positive slow twitch fibers in both WT and *dy*^*2J*^*/dy*^*2J*^ animals. Even though metformin treatment tended to increase expression of MyHC, type 1, there were no significant differences between any of the four studied groups (Fig. 8B).

### Metformin treatment enhances the weight of white adipose tissue in *dy*^*2J*^*/dy*^*2J*^ mice

**Figure 9:**
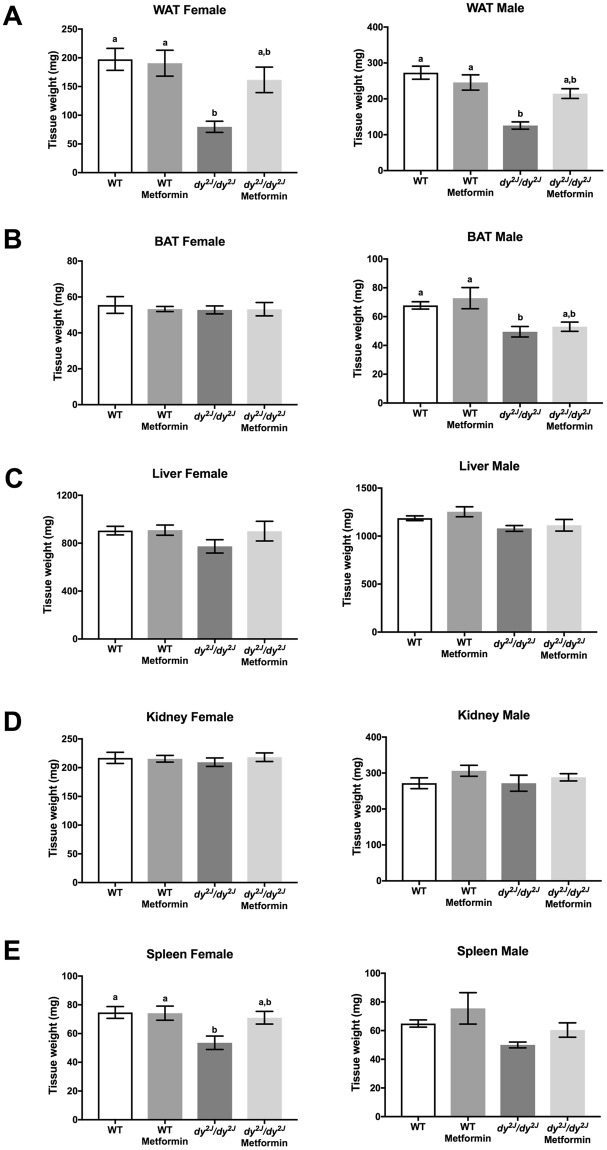
Tissue weights. (**A**) White perigenital adipose tissue (WAT). (**B**) Brown intercostal adipose tissue (BAT). (**C**) Liver. (**D**) Kidney. (**E**) Spleen. Values are expressed in grams (collected after four weeks of treatment). WT control: females = 7, males = 13; WT metformin: females = 10, males = 6; *dy*^*2J*^*/dy*^*2J*^ control: females = 5, males = 4; *dy*^*2J*^*/dy*^*2J*^ metformin: females = 5, males = 5. Results are expressed as mean ± SEM. Statistical significance was assessed by one-way ANOVA followed by Bonferroni *post hoc* test. *p* < 0.05 values were considered as statistically significantly different. Letters a and b were used to express the differences among groups and columns with the same letter are not significantly different.

Finally, to investigate whether metformin influenced non-muscle organs we analyzed the weights of white adipose tissue, brown adipose tissue, liver, kidney and spleen. In *dy*^*2J*^*/dy*^*2J*^ mice we noted a 60% and 54% decrease of white adipose tissue weight in females and males, respectively, and metformin treatment enhanced *dy*^*2J*^*/dy*^*2J*^ white adipose tissue weight in both females and males but did not impact WT white adipose tissue weight (Fig. 9A). The weight of brown adipose tissue was not affected in *dy*^*2J*^*/dy*^*2J*^ females but was significantly reduced in *dy*^*2J*^*/dy*^*2J*^ males. Metformin did not alter the weight of brown adipose tissue in female mice but marginally increased the brown adipose tissue weight in *dy*^*2J*^*/dy*^*2J*^ males (Fig. 9B). Neither liver nor kidney weights were affected in *dy*^*2J*^*/dy*^*2J*^ females and males (Fig. 9C,D). Spleen weight, on the other hand, was reduced by 20% in *dy*^*2J*^*/dy*^*2J*^ females (a non-significant reduction was also noted in *dy*^*2J*^*/dy*^*2J*^males) and metformin slightly increased spleen weight in *dy*^*2J*^*/dy*^*2J*^ females (Fig. 9E).

## Discussion

LAMA2-CMD, the second-most common form of congenital muscular dystrophy, remains incurable despite the development of successful genetic and pharmacological preclinical treatment strategies^1,2^. Metformin is FDA approved for treatment of type II diabetes in children over 10 years of age but is also used off-label to treat obesity in adolescents^17^. In this study, we show that metformin enhances weight gain, restores energy efficiency, augments muscle function, improves morphological features of muscular dystrophy and reduces the expression of some fibrosis-related genes in *dy*^*2J*^*/dy*^*2J*^ females (and to a lesser extent in *dy*^*2J*^*/dy*^*2J*^ males). Some features were not improved, such as exploratory locomotion but this may be due to the fact that metformin had no effect on hindleg lameness. Peripheral neuropathy is particularly evident in *dy*^*2J*^*/dy*^*2J*^ mice but is not a clinical manifestation in patients^18^. Another therapeutic candidate for LAMA2-CMD is losartan that was demonstrated to confer clinical improvement and reduce fibrosis in the *dy*^*2J*^*/dy*^*2J*^ mouse model^19^. The anti-apoptotic compound omigapil also provided beneficial effects in *dy*^*2J*^*/dy*^*2J*^ mice^20^ as well as in the *dy*^*W*^*/dy*^*W*^ mouse^21^ (another mouse model of LAMA2-CMD). Losartan and omigapil are expected to target fibrosis and apoptosis, respectively, but neither compound has been shown to regulate metabolism, which is significantly altered in LAMA2-CMD as well as in other muscular dystrophies^5,22,23^.

Metformin has been described to control metabolism, for example by decreasing lipogenesis and gluconeogenesis and modulating mitochondrial function^24^. Studies demonstrate that metformin increases healthspan and lifespan in mice^25^ and in elderly patients, improving glucose tolerance and regulating expression not only of metabolic genes but also collagen and DNA repair-related-genes in muscle and adipose tissue^7^. Moreover, it was recently described that metformin treatment attenuated fibrosis and insulin resistance in adipose tissue caused by doxorubicin treatment in rats^26^. In skeletal muscle, metformin treatment improved the oxidative metabolism in gastrocnemius muscle from mice and reduced muscle injury induced by cardiotoxin^9^. In the same study, reduction of cell damage and necrosis was demonstrated *in vitro* using C2C12 myotubes subjected to metformin treatment^9^. Finally, in tumor-bearing animals metformin was shown to increase protein synthesis and reduces protein degradation in gastrocnemius muscle^27^. It is plausible that metformin improves oxidative metabolism, which is significantly impaired in LAMA2-CMD cells^5^. On the other hand, while metformin conferred positive effects in *mdx* skeletal muscle, no clear protective actions on dystrophic metabolism were observed^8^. Hence, the mechanisms of metformin action in *dy*^*2J*^*/dy*^*2J*^ mice are still to be clarified. Yet, it is interesting to note that another compound (N-acetyl-cysteine), which affects metabolism by reducing the formation of reactive oxygen species that may arise through insufficient mitochondrial respiration, also improves muscle function and structure in *dy*^*2J*^*/dy*^*2J*^ mice (Harandi *et al*., unpublished data). Nevertheless, there are some other limitations of this study. For example, treatment was initiated at 6-weeks of age when muscle impairment is already readily detected in *dy*^*2J*^*/dy*^*2J*^ mice^14^. It would be interesting to start metformin treatment sooner and continue over a longer period of time (as no overt side effects of metformin were noted). Moreover, we have only analysed histology of one skeletal muscle (quadriceps).

It is well known that gender affects a wide variety of physiological functions including cardiovascular and autoimmune systems and influences a broad range of diseases such as gastrointestinal, liver, kidney, endocrine, blood and neurological disorders. Moreover, gender also impacts pharmacokinetics and pharmacodynamics^28^. Duchenne muscular dystrophy is an X-linked recessive muscular dystrophy, thus affecting only boys, but apart from gender differences due to the genetic inheritance pattern, there is only little data available regarding gender differences in disease progression of muscular dystrophy^29–31^. Bearing this in mind, we monitored the disease progression during four weeks in 6-week-old *dy*^*2J*^*/dy*^*2J*^ females and males. We found several important gender differences (at least in this age range). Weight gain was lower in *dy*^*2J*^*/dy*^*2J*^ female mice compared to *dy*^*2J*^*/dy*^*2J*^ males; water intake was significantly reduced in *dy*^*2J*^*/dy*^*2J*^ females compared to WT females (not in corresponding males); a larger decrease of relative grip strength was noted in *dy*^*2J*^*/dy*^*2J*^ females compared to *dy*^*2J*^*/dy*^*2J*^ males and the shift in fiber size distribution was much more pronounced in *dy*^*2J*^*/dy*^*2J*^ females compared to males. Thus, to our knowledge, this is the first time gender differences are reported in LAMA2-CMD.

Energy efficiency is known as the balance between energy intake and expenditure and is determinant to muscle mass maintenance^15,32,33^. Factors such as increased resting energy expenditure and uncoupled systems can lead to a metabolic inefficiency and consequently muscle loss, independently of energy intake^33^. It is known that metformin activates AMP-activated protein kinase, a critical sensor in the cell that regulates cellular metabolism, leading to an increase of catabolic pathways to produce ATP and a decrease of anabolic pathways that consume ATP, equilibrating the energy stores in cells^24,34,35^. In muscular dystrophies, food and drink ingest can be impaired by dysphagia and tend to worsen during disease progression^36–39^. Our data shows that even though food intake was not altered in *dy*^*2J*^*/dy*^*2J*^ female and male mice, the energy efficiency was drastically decreased, being most prominent in females with negative values compared to WT mice (which we considered 100% efficient). Notably, metformin was able to improve energy efficiency by 60% in *dy*^*2J*^*/dy*^*2J*^ females and 20% in *dy*^*2J*^*/dy*^*2J*^ males, again supporting the beneficial effects of treatment by gender.

In conclusion, this study shows that metformin treatment significantly reduces muscular dystrophy in *dy*^*2J*^*/dy*^*2J*^ females. Importantly, metformin is already approved for use in humans, which is advantageous from a clinical point of view. Nevertheless, metformin treatment does not target the primary genetic defect and is not expected to completely cure LAMA2-CMD. Yet, metformin could be used as a supportive treatment that may improve many of the pathological symptoms in LAMA2-CMD.

## Materials and Methods

### Animals

Heterozygous *dy*^*2J*^*/dy*^*2J*^ (B6.WK-Lama2dy-2J/J) were obtained from Jackson Laboratory and bred and maintained in our animal facility to generate *dy*^*2J*^*/dy*^*2J*^ and WT mice according to institutional animal care guidelines. All experimental procedures involving animals were approved by the Malmö/Lund (Sweden) ethical committee for animal research (ethical permit number 5.8.18–02255/2017) in accordance with guidelines issues by the Swedish Board of Agriculture. The animals were maintained at 22 ± 2 °C with a regular light-dark cycle (light on from 6:00 am to 6:00 pm) and had free access to food and water. The diet consisted of 51.2% carbohydrate, 22% protein and 4.25% fat (Special Diet Services). Six-week-old mice were separated according to gender and into wild-type (WT) and dystrophic (*dy*^*2J*^*/dy*^*2J*^) groups and further randomly subdivided into WT control (7 females and 13 males), WT metformin-treated (10 females and 6 males), *dy*^*2J*^*/dy*^*2J*^ control (5 females and 4 males) and *dy*^*2J*^*/dy*^*2J*^ metformin-treated (5 females and 5 males) groups. Thus, mice were housed according to genotype, gender and treatment. Mouse numbers vary slightly for the different outcome measures and we refer to further information in the figure legends.

### Treatment

Metformin (CAS 1115-70-4, Calbiochem) diluted in filtered water was administrated by oral gavage once a day at 250 mg/kg body weight during four weeks. Control animals received filtered water by oral gavage. Initial and final body weight and weight gain were analyzed. Food and water intake were estimated twice-a-week (by weight measurements divided by number of animals in cage). The energy efficiency ratio was calculated as the weight gain (g) during the experimental period divided by the cumulative energy intake over the same period (kcal), according to Jung *et al*.^15^.

### Locomotor activity and grip strength

After four weeks of treatment, animals were subjected to exploratory locomotion testing and grip strength analysis. Exploratory locomotion was evaluated in an open-field test, as previously described^40^. In each experiment, a mouse was placed into a new cage and allowed to explore the cage for five minutes. The time that the mouse spent moving around was measured.

Forelimb grip strength was measured using a grip-strength meter (Columbus Instruments) as previously described^40^. In brief, the mouse was held by the base of the tail and allowed to grasp the flat wire mesh of the pull bar with its forepaws. When the mouse got a good grip, it was slowly pulled away by its tail until it released the pull bar. Each mouse was allowed to pull the pull bar five times. The two lowest values were rejected and the mean of the three remaining values was counted. Animals were not subjected to any training prior to the experiment. Grip strength was calculated as force divided by final body weight^41^.

### Tissue collection

Mice were sacrificed by cervical dislocation. Tissues were rapidly excised, carefully dissected, and weighed. Skeletal muscles isolated were soleus, gastrocnemius, tibialis anterior and quadriceps. Heart, white adipose tissue (perigenital), brown adipose tissue (intercostal), liver, spleen and kidney were isolated and weighed as well.

### Histology and immunohistochemistry

For morphometric analyses, quadriceps muscles were either embedded in OCT compound (Tissue-Tek) and frozen in liquid nitrogen or embedded in paraffin. Paraffin-embedded specimens were sectioned using a microtome (5 μm) (Microm H355) and OCT embedded sections were sectioned using a cryostat (7 μm) (Microm HM 560). Paraffin sections were stained with hematoxylin and eosin (H&E) staining and cryosections were subjected to immunostaining (see further down). H&E stained sections were scanned using an Aperio ScanScope CS2 scanner with ScanScope console version 8.2.0.1263. Central nucleation was quantified using ImageJ software version 1.43 u, Cell Counter plug-in (NIH). The whole quadriceps cross-section muscle was used for quantification and the percentage of central nuclei was subsequently calculated. The fiber area of biotinylated wheat germ agglutinin (WGA) stained muscle fibers was measured and quantified using ImageJ and approximately 300 fibers per mouse were analyzed.

Immunohistochemistry was performed as previously described^42^ using a monoclonal antibody against anti-myosin (skeletal, slow; M8421, Sigma Aldrich). In addition, biotinylated WGA was used as a membrane marker. The secondary antibody was goat anti-mouse IgG 546 (Thermo Fisher Scientific) together with avidin. The slides were analyzed by Zeiss Axioplan fluorescence microscope (Zeiss) and images were captured using an ORCA 1394 ER digital camera (Hamamatsu Photonics) and Openlab software version 4 (Improvision). The whole quadriceps cross-section muscle was used for quantification and the percentage of fibers with positive MyHC type 1 staining was subsequently calculated (by visual assessment).

### Sirius red/fast green quantification

Collagen content was quantified by a colorimetric method as previously described^43^. Around 15 paraffin sections (15 μm) were placed in a plastic tube. Paraffin removal was accomplished by immersing the sections in xylene (5 min), xylene/ethanol (1:1) (5 min), ethanol (5 min), ethanol/water (1:1) (5 min) and water (5 min). The sections were then stained with fast green/sirius red for 30 minutes on rotation and subsequently washed with distilled water until excess dye was removed and solution was clear. One ml of 0.1 N NaOH was added and the solutions were analyzed by absorbance at 560 nm and 605 nm.

### Real time PCR analysis

RNA isolation was performed by using RNeasy Fibrous Tissue Kit QIAGEN according to manufacturer’s recommendations. First-strand cDNA was synthesized from total RNA (0.8 μg) with oligonucleotide dT15 primers and random primers p(dN)6 by use of First Strand cDNA synthesis kit (Roche). Real time-PCRs were performed using Light Cycler 480 SYBR Green Master I (Roche) and were analyzed by Light Cycler 480 SW 1.5 software (Roche). Oligonucleotide sequences used for PCR are listed in Supplementary Table S1. Primers were from Sigma (KiCqStart SYBR Green Primers) or designed as previously described^44^ using Primer3 software (http://primer3plus.com/cgi-bin/dev/primer3plus.cgi, last accessed, Aug 10, 2018). Primer parameters were defined as follows: product size: 50–150 bp; primer size: 18–22 bp (opt: 20); primer Tm: 57–63 °C (opt: 60); primer GC%: 40–60%; maximum self-complementarity: 3–4. The Operon tool (http://www.operon.com/tools/oligo-analysis-tool.aspx, last accessed Aug 10, 2018) was utilized to check the putative primer-dimer formation. Amplification conditions consisted of 5 s of denaturation at 94 °C, 9 s of annealing at 55–60 °C and 9 s of extension at 72 °C for each step for 45 cycles. The relative amount of all mRNAs was calculated using the comparative CT method. *Rplp0* was used as the invariant control.

### Statistical analysis

All data are shown as mean ± S.E.M. Statistical analysis of the data was performed by means of two-way analysis of variance (ANOVA) with Bonferroni *post hoc* test for comparison of gender and disease effect and one-way analysis of variance (ANOVA) with Bonferroni *post hoc* test for comparison of treatment effect. p < 0.05 values were considered as statistically significant.

## Electronic supplementary material

Supplementary information

## Data Availability

The datasets generated during and/or analyzed during the current study are available from the corresponding authors on reasonable request.
