# Supplementary material for: Effects of metformin on congenital muscular dystrophy type 1A disease progression in mice: a gender impact study
Source: Sci Rep. 2018 Nov 2;8:16302. doi: 10.1038/s41598-018-34362-2 (PMC6214987; doi:10.1038/s41598-018-34362-2)
Supplement: Supplementary file 1 — Supplementary information [file 41598_2018_34362_MOESM1_ESM.pdf]

Supplementary information

Effects of metformin on congenital muscular dystrophy type 1A disease progression in mice: a gender impact study

Cibely C. Fontes-Oliveira, Bernardo Moreira Soares Oliveira, Zandra Körner, Vahid M. Harandi and Madeleine Durbeej

Supplementary Figure 1

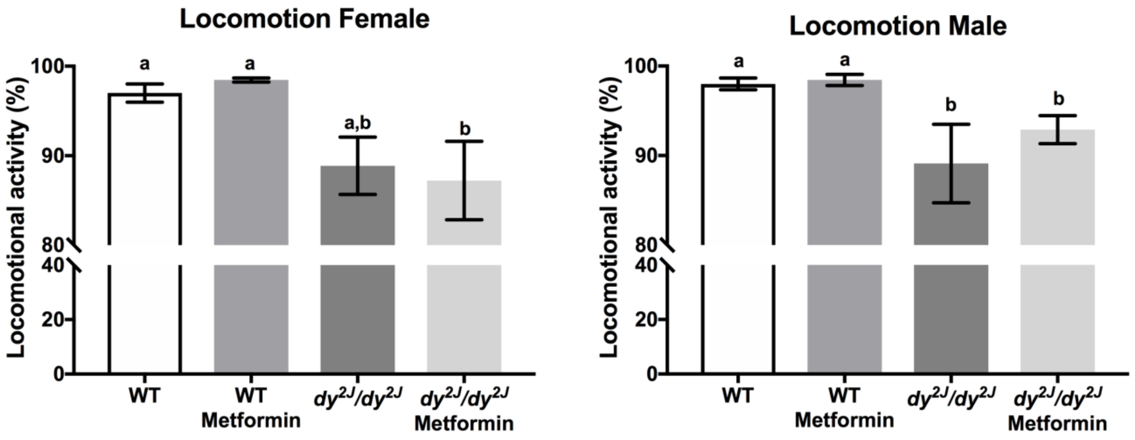

Administration of metformin does not improve the exploratory locomotion of dy^{2J}/dy^{2J} mice in an open-field test. WT control: females=5, males=8; WT metformin: females=8, males=6; dy^{2J}/dy^{2J} control: females=5, males=3; dy^{2J}/dy^{2J} metformin: females=6, males=6. Results are expressed as mean ± SEM. Statistical significance was assessed by one-way ANOVA followed by Bonferroni *post hoc* test. $p<0.05$ values were considered as statistically significantly different. Letters a and b were used to express the differences among groups and columns with the same letter are not significantly different.

Supplementary Table 1. Primer sequences used for real time-PCR analysis.

Name	Left (5'-3')	Right (5'-3')	Source
Fn1	CCTATAGGATTGGAGACACG	GTTGGTAAATAGCTGTTCGG	KiCqStart®
Col3a1	ACTCAAGAGTGGAGAATACTG	AACATGTTTCTTCTCTGCAC	KiCqStart®
Tgfb1	CTATACAGCACAGTATGCAAG	CATCTGTAATGTTGAACTGGG	KiCqStart®
MuRF1	GAAGTGTGCCAACGACATCT	ACGGAAACGACCTCCAGAC	Designed
MAFbx	TGTGCGATGTTACCCAAGAA	GGTGAAAGTGAGACGGAGCA	Designed
Pgc1α	AAGGTCCCCAGGCAGTAGAT	TTCAGACTCCCGCTTCTCAT	Designed
Rplp0	ACATCTCCCCCTTCTCCTTCGG	GTTGCGGACACCCTCCAGAAAG	Designed

Abbreviations: *Fn1*: fibronectin; *Col3a1*: collagen, type III, α1; *Tgfb1*: transforming growth factor β1; *MuRF1*: muscle RING-finger protein-1; *MAFbx*: muscle atrophy F-box protein; *Pgc1α*: peroxisome proliferator-activated receptor gamma coactivator 1α; *Rplp0*: acidic ribosomal phosphoprotein P0. Source: Designed using primer3 software or purchased by Sigma (KiCqStart predesigned primers).
